# ANXA11 regulates the tumorigenesis, lymph node metastasis and 5-fluorouracil sensitivity of murine hepatocarcinoma Hca-P cells by targeting c-Jun

**DOI:** 10.18632/oncotarget.7484

**Published:** 2016-02-18

**Authors:** Shuqing Liu, Chunmei Guo, Jiasheng Wang, Bo Wang, Houbao Qi, Ming-Zhong Sun

**Affiliations:** ^1^ Department of Biochemistry, Dalian Medical University, Dalian 116044, China; ^2^ Department of Biotechnology, Dalian Medical University, Dalian 116044, China; ^3^ Department of Pathology, Dalian Medical University, Dalian 116044, China

**Keywords:** Anxa11, hepatocarcinoma, lymphatic metastasis, chemoresistance, c-Jun

## Abstract

Annexin A11 (Anxa11) is associated with various cancers. Using a pair of syngeneic murine hepatocarcinoma cells, Hca-P with ~25% and Hca-F with ~75% lymph node metastatic (LNM) potentials, we demonstrated Anxa11 involvement in hepatocarcinoma lymphatic metastasis. Here, ANXA11 acted as a suppressor for the tumorigenicity, LNM and 5-FU resistance of Hca-P *via* c-Jun. We constructed monoclonal Hca-P cell line with stable ANXA11 knockdown. Although Bax and Bcl-2 levels increased in shRNA-Anxa11-transfected Hca-P, ANXA11 downregulation showed no clear effect on Hca-P apoptosis. ANXA11 downregulation promoted *in vitro* migration and invasion capacities of Hca-P. *In situ* adhesion potential of Hca-P cells toward LN was significantly enhanced following ANXA11 downregulation. Consistently, ANXA11 downregulation promoted the *in vivo* tumor growth and LNM capacities of Hca-P cells. ANXA11 knockdown enhanced the chemoresistance of Hca-P cells specifically toward 5-FU instead of cisplatin. Its downregulation increased c-Jun (pSer73) and decreased c-Jun (pSer243) levels in Hca-P. c-Jun (pSer243) downregulation seemed to be only correlated with ANXA11 knockdown without the connection to 5-FU treatment. Interestingly, compared with scramble-Hca-P cells, the levels of c-Jun and c-Jun (pSer73) in shRNA-Anxa11-Hca-P cells were upregulated in the presences of 0.1 and 1.0 mg/L 5-FU. The levels changes from c-Jun and c-Jun (pSer73) in Hca-P cells showed a more obvious tendency with the combination of ANXA11 knockdown and 5-FU treatment. ANXA11 level regulates LNM and 5-FU resistance of Hca-P *via* c-Jun pathway. It might play an important role in hepatocarcinoma cell malignancy and be a therapeutic target for hepatocarcinoma.

## INTRODUCTION

Hepatocellular carcinoma (HCC) is one of the most common cancers worldwide [1, 2]. The lymphatic metastasis and chemoresistance are the major problems leading to its high recurrence with low post-surgical 5-years’ survival [3–5]. Tumor metastasis is a multistep process, including the invasion of extracellular matrix (ECM), intravasation, translocation, migration and invasion of a secondary site, and finally the formation of metastatic nodules [3,5]. Deep study on the metastatic mechanisms can get the novel therapeutic targets and improve the prognosis for HCC.

Annexin A11 (Anxa11) is one member of annexins that are Ca^2+^-regulated phospholipid-binding proteins [6–10]. Like other annexins, the C-terminus of Anxa11 contains homologous tetrad annexin repeat core with Ca^2+^ binding sites. Ca^2+^ is competently and duly required for membrane binding, thermostability and tertiary structure of ANXA11. The N-terminus of Anxa11 is rich in glycine, tyrosine and proline residues with calcyclin (S100A6) and apoptosis-linked gene-2 protein (ALG-2) binding sites [11–13]. Anxa11 plays an important role in cell division, differentiation, apoptosis, vesicle trafficking and Ca^2+^ signaling. Anxa11 dysregulation is involved in the progression, drug-resistance, recurrence of systemic autoimmune disease and cancer [14–18].

c-Jun is a central component of transcription factor complex activator-protein 1 (AP-1) and a substrate of c-Jun-N-terminal kinases (JNKs) [19, 20]. c-Jun and its phosphorylation form p-c-Jun play important roles in cancer cell differentiation, invasion, metastasis, apoptosis, and chemoresistance through the action with other genes [21–24]. No study was reported on the association of c-Jun with the lymphatic metastasis or chemoresistance in HCC.

Hca-P with low lymph node metastatic (LNM) potential (~25%) and Hca-F with high LNM potential (~75%) were established in our research group as a pair of synogenetic murine HCC ascites cell lines with same genetic background [25–35]. They show the advantage of metastasizing specifically to LN without disseminating to other organs. They are the ideal experimental subjects for investigating the “pure” underlying LNM in the malignant hepatocellular tumors deriving from epithelia [5, 25-27, 32, 34-38]. We previously reported the protein expression level of ANXA11 in Hca-P was 2-fold higher than Hca-F cells [39], suggesting ANXA11 might act as a potential suppressor for the lymphatic metastasis of murine HCC.

In this work, we investigated the effect of ANXA11 expression level on the malignant properties and underlying mechanism of Hca-P cells. Stable ANXA11 knockdown significantly enhanced the *in vitro* migration and invasion of Hca-P cells. ANXA11 downregulation also promoted the *in vivo* lymph node metastatic capacities of Hca-P cells. ANXA11 level regulated the lymphatic metastasis and 5-FU chemoresistance of Hca-P cells *via* c-Jun pathway.

## RESULTS

### ANXA11 is stably downregulated in its monoclonal shRNA-transfected Hca-P cells

**Figure 1:**
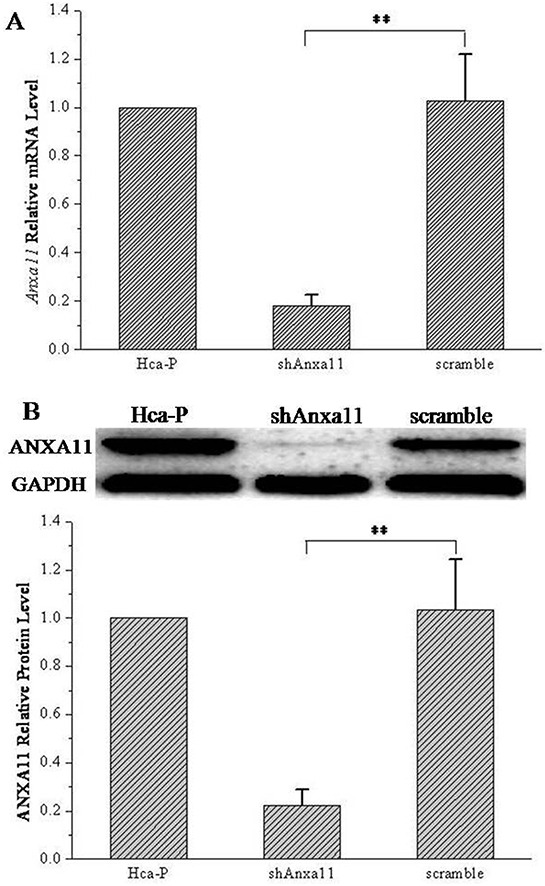
Anxa11 knockdown by RNAi **A.** Relative *Anxa11* mRNA levels in Hca-P, shAnxa11- Hca-P and scramble-Hca-P cells were determined by qRT-PCR using GAPDH as internal reference. **B.** WB assay of ANXA11 levels in Hca-P, shAnxa11-Hca-P and scramble-Hca-P cells. GAPDH was the internal reference. Triplicate independent measurements were performed for WB assays. No statistical significances for the differences between Hca-P and scramble-Hca-P cells at both mRNA and protein levels for Anxa11. ** Refers to the difference is of statistical significance (*P* < 0.01).

Hca-P cells transfected with the specific shRNA of *Anxa11* and with the shRNA of unrelated targeting sequence were named as shAnxa11-Hca-P and scramble-Hca-P cells. The monoclonal shAnxa11-Hca-P and scramble-Hca-P cells were obtained by limited dilution against G418 screening. qRT-PCR and WB showed *Axna11* mRNA and ANXA11 protein levels were decreased by 82.49±3.49% (*P* < 0.01, Figure 1A) and 80.53±4.06% (*P* < 0.01, Figure 1B) in shAnxa11-Hca-P cells compared with scramble-Hca-P cells, while no difference was detected for its expression levels between scramble-Hca-P and Hca-P cells. The establishment of monoclonal shAnxa11-Hca-P cells with stable ANXA11 downregulation provided solid material for further study on the potential role of ANXA11 in murine HCC lymphatic metastasis.

### ANXA11 downregulation shows no clear effect on Hca-P cell apoptosis

**Figure 2:**
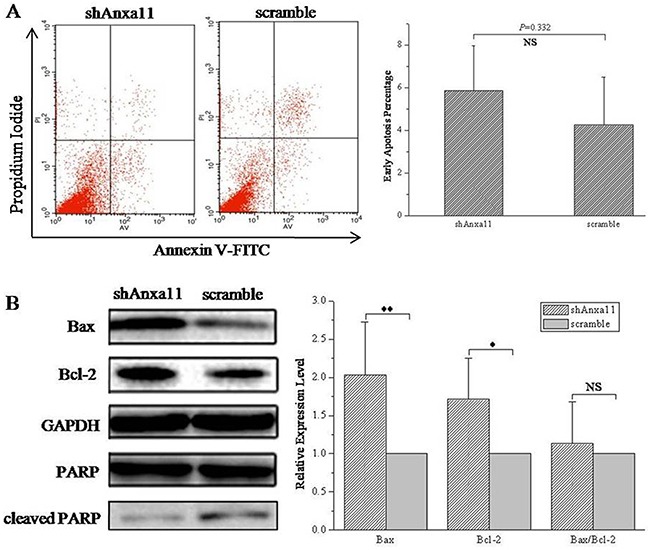
Anxa11 knockdown on Hca-P apoptosis **A.** Flow cytometry showed Anxa11 level had no clear effect on Hca-P apoptosis (*P*>0.05). **B.** WB assay of the levels of Bax, Bcl-2, PARP and cleaved PARP in shAnxa11-Hca-P and scramble-Hca-P cells. Anxa11 knockdown shows no clear effect on the levels of PARP and cleaved PARP. Bax (** *P* <0.01) and Bcl-2 (* *P*<0.05) were up-regulated with statistical significances in shAnxa11-Hca-P compared with scramble-Hca-P cells. Anxa11downregulation exhibited no influence on Bax/Bcl-2 ratio (*P*>0.05). Three independent measurements were performed for each protein molecule.

ANXA11 knockdown exhibits no effect on apoptosis of Hca-P cells. The influence of ANXA11 downregulation on Hca-P cell apoptosis was detected by flow cytometry and WB. Flow cytometry results (Figure 2A) showed there was no difference between the apoptosis rate of shAnxa11-Hca-P (5.87±2.10%) cells and scramble-Hca-P (4.24±2.25%) cells (*P*>0.05). No statistical significant difference was determined for the expression levels of poly adeno ribose polymerase (PARP) and cleaved PARP in Hca-P cells following the knockdown of ANXA11 (Figure 2B). Although the levels of Bax and Bcl-2 were upregulated with statistical significances with *P* <0.01 and *P* <0.05 (Figure 2B) in shAnxa11-Hca-P compared with scramble-Hca-P cells, ANXA11 knockdown did not alter the expression level ratio of Bax/Bcl-2 (*P*>0.05, Figure 2B).

### ANXA11 knockdown promotes *in vitro* migration, invasion, *in situ* LN adhesion potential of Hca-P cells

**Figure 3:**
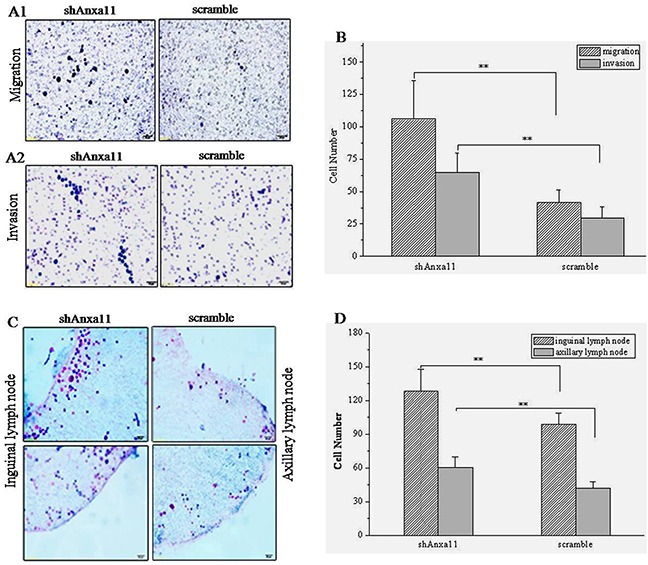
Influence of Anxa11 downregulation on *in vitro* migration, invasion and *in situ* LN adhesion potentials of Hca-P cells **A.** and **B.** Anxa11 downregulation significantly enhanced the migration ability **A1.** and invasion capacity **A2.** of Hca-P cells, **P<0.01. **C.** and **D.** Anxa11 downregulation significantly promoted *in situ* inguinal and axillary LNs adhesion capacities of Hca-P cells, **P<0.01. Three independent measurements were performed for migration, invasion and LN adhesion assays.

We reported ANXA11 linked to hepatocarcinoma lymphatic metastasis as its level was 2-fold higher in Hca-P than Hca-F cells [39]. The stable knockdown of ANXA11 on *in vitro* migration, invasion and *in situ* adhesion capacity to LN of Hca-P cells was performed. As shown in Figure 3, the numbers of migrated (106.0±29.7, *P*<0.01; Figure 3A1 and 3B) and invaded (64.7±15.0, *P*<0.01; Figure 3A2 and 3B) shAnxa11-Hca-P cells increased to ~253.6% and 217.8% of the numbers of 41.8±9.3 and 29.7±8.4 for scramble-Hca-P cells. ANXA11 knockdown also improved the *in situ* LN adhesion potential of Hca-P cells. shAnxa11-Hca-P cells showed a greater adhesive potential to inguinal and axillary LNs than scramble-Hca-P cells (Table 1). As the results shown in Figure 3C and 3D, the numbers of shAnxa11-Hca-P cells adhered to inguinal and axillary LNs were measured as128.4±19.4 and 98.8±10.1 that were 2.1- and 2.4-folds of 60.6±9.5 and 42.0±6.0 for scramble-Hca-P cells with statistical significances (*P<0.01*). The above results indicate ANXA11 downregulation enhances the migration, invasion and adhesive capacities of Hca-P, which might result in its enhanced lymphatic metastasis malignancy.

**Table 1:** ANXA11 knockdown on *in situ* adhesion ability of Hca-P cells to lymph node

Cell	LN	Cell number					Mean number
		Field 1	Field 2	Field 3	Field 4	Field 5	
shAnxa11-Hca-P	Inguinal	138^a^	124	156	119	105	128.4±19.4
	Axillary	113	91	96	105	89	98.8±10.1
scramble-Hca-P	Inguinal	70	46	58	61	68	60.6±9.5
	Axillary	41	36	37	46	50	42.0±6.0

a Note: Refers to the averaged number of adhered cells from three assays. Adhesion potential differences to inguinal and axillary LNs between shAnxa11-Hca-P and scramble-Hca-P cells were of statistical significance (P<0.01).

### ANXA11 stable knockdown promotes *in vivo* tumorigenicity and LNM of Hca-P cells

**Figure 4:**
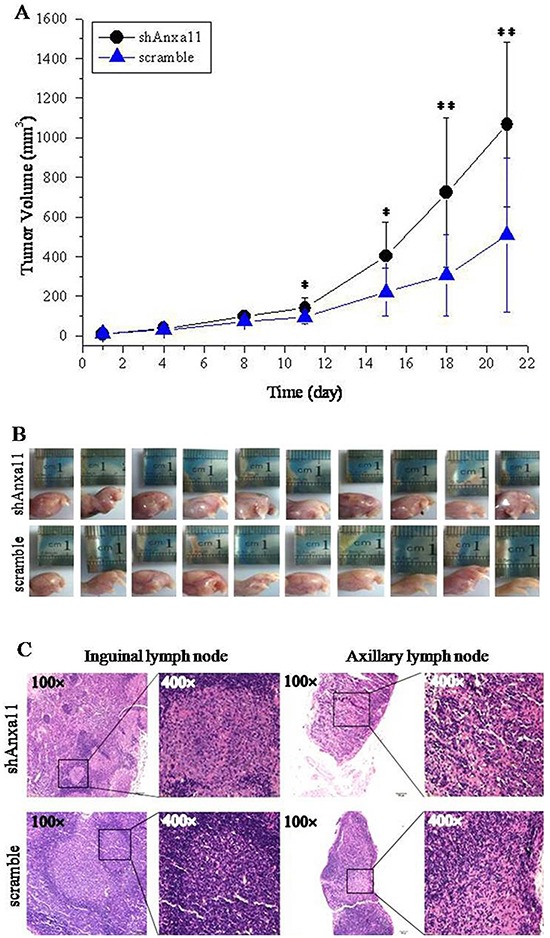
Anxa11 knockdown on tumorigenicity and LNM capacity for Hca-P cells **A.** shAnxa11-Hca-P-transplaneted mice showed increased tumor growth speed than scramble-Hca-P-transplanted ones. Results were represented as the mean tumor volume ± SD of each group. The tumor volume differences were with *P*<0.05 on the 11^th^ and 15^th^ days, and with *P*<0.01 on the 18^th^ and 21^st^ days after cell inoculation. **B.** Images of the primary tumors formed on the footpads of mice on the 21^st^ day after cell inoculations. **C.** Photos of inguinal and axillary LNs from shAnxa11-Hca-P-tanspanted mice and scramble-Hca-P-transplanted mice taken at the magnifications of 200× (left) and 400× (right).

ANXA11 downregulation effect on tumorigenicity of Hca-P cells was investigated. shAnxa11-Hca-P and scramble-Hca-P cells were transplanted into the left footpads of mice. The sizes and volumes of the primary solid tumors formed on mice footpads were measured at 1, 4, 8, 11, 15, 18 and 21 days following cell inoculation. As the results showed in Figure 4A, ANXA11 knockdown exhibited promotion tendency to the volume of formed tumors (Figure 4A). The increase of tumor volumes showed statistical significances with *P*<0.05 in 11 and 15 days, and *P*<0.01 in 18 and 21 days for shAnxa11-Hca-P-transplanted mice compared with scramble-Hca-P-transplanted mice due to Anxa11 downregulation (Figure 4A). Figure 4B showed the images of the primary tumors after cell inoculations for 21 days, the tumor sizes of shAnxa11-Hca-P-transpalnted mice were bigger than scramble-Hca-P-transplanted ones. Consequently, there was a mass difference with stastical significance of the primary tumors formed on mouse footpad between the two group cells. After cell inoculations for 21 days, the masses for collected primary tumors from 10 shAnxa11-transplanted mice were 2.1330, 1.6022, 1.7563, 1.7020, 1.6747, 0.3022, 1.0448, 1.2866, 1.7573 and 0.7332 g with an averaged mass of 1.3992±0.5566 g. The masses of 9 (1 was lost when dissected it from footpad) scramble-Hca-P-transplanted mice were measured as 0.7403, 1.2820, 1.4073, 1.0899, 0.5000, 0.0779, 1.063, 0.9171 and 0.1423 g with an average of 0.8022±0.4764 g. shAnxa11-transplanted mice showed ~74.4% increased primary tumor mass than scramble-Hca-P-transplanted ones with stastical significance (*P*=0.02309). ANXA11 downregulation inversely correlated *in vivo* tumor formation of Hca-P cells.

Stable ANXA11 knockdown promoted the LNM of Hca-P cells. We found the number of LNs with invaded shAnxa11-Hca-P cells were more than that of LNs invaded with scramble-Hca-P cells. 2/6 and 6/6 collected inguinal and axillary LNs were with invaded shAnxa11- Hca-P and scramble-Hca-P cells (*P*<0.01). The structure of shAnxa11-Hca-P-metastasized LN was disorganized, while, in contrast, scramble-Hca-P-metastasized LN still retained in a relatively complete structure, as shown in Figure 4C. ANXA11 downregulation promoted the *in vivo* LNM capacity of Hca-P cells.

### ANXA11 knockdown enhances the chemoresistance of Hca-P cells to 5-FU

**Figure 5:**
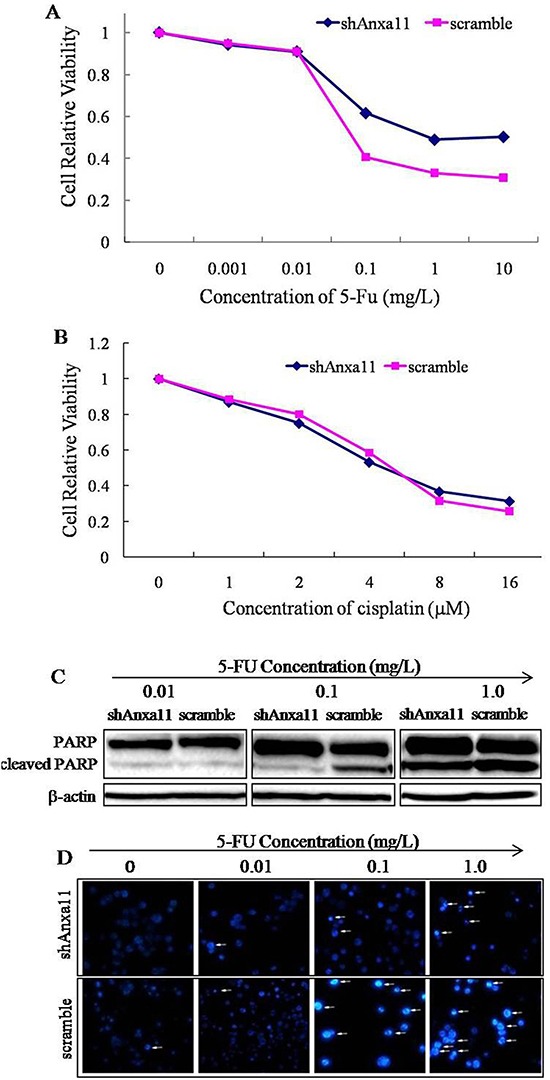
Anxa11 knockdown influence on Hca-P cell drug sensitivity to 5-FU and cisplatin Dose-response curves for shAnxa11-Hca-P and scramble-Hca-P cells to 5-FU **A.** and cisplatin **B.** treatments. Cell viability was determined by CCK-8 assay. The viability differences between the two cell lines at 0.1, 1.0 and 10 mg/L 5-FU administrations were of statistical significances (*P*<0.01). **C.** Triplicate WB assays of the levels of cleaved PARP with the treatments of different concentrations of 5-FU in shAnxa11-Hca-P and scramble-Hca -P cells. **D.** Hoechst 33258 staining assay for shAnxa11-Hca-P and scramble-Hca-P cells with the treatments of different concentrations of 5-FU.

5-FU and cisplatin are adjuvant drugs in the clinical treatment of HCC patients. CCK-8 assay was performed to investigate the influences of ANXA11 level on the drug sensitivity of Hca-P cells to 5-FU (Figure 5A) and cisplatin (Figure 5B). No clear influence on Hca-P sensitivity to cisplatin administration (Figure 5B). In 5-FU concentration ranged in 0.01 to 10 mg/L, the viabilities of shAnxa11-Hca-P and scramble-Hca-P cells decreased dose-dependently (Figure 5A). shAnxa11-Hca-P showed a decreased sensitivity to 5-FU treatment than scramble-Hca-P cells (Figure 5A, *P*<0.01).

The influence of ANXA11 knockdown on the cell apoptosis induced by 5-FU was evaluated by Hoechst 33258 staining assay by determining the expression level change of cleaved PARP in Hca-P cells. Compared with the scramble-Hca-P cells, WB result indicated that the level of cleaved PARP induced by 5-FU administration was significantly reduced in shAnxa11-Hca-P cells following ANXA11 downregulation (Figure 5B). ANXA11 stable knockdown passivates the sensitivity of Hca-P cells to 5-FU-induced apoptosis (Figure 5C). Hoechst 33258 staining assay demonstrated that the condensed and fragmented nuclei of apoptotic cells were highly decreased in shAnxa11-Hca-P cells in comparison with scramble- Hca-P cells with the treatments of same concentrations of 5-FU (Figure 5C). ANXA11 downregulation enhances Hca-P cell chemoresistance to 5-FU specifically instead of cisplatin, which provides rational clinical treatment clue for HCC. It may be of potential application in chemotherapy of HCC patients.

### ANXA11 downregulation promotes Hca-P LNM and 5-FU resistance *via* c-Jun pathway

**Figure 6:**
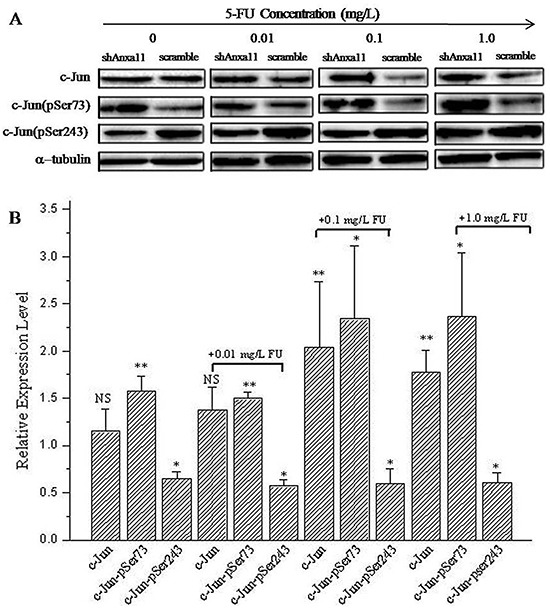
ANXA11 is linked to c-Jun pathway on Hca-P sensitivity to 5-FU **A.** WB images of c-Jun, c-Jun (pSer73) and c-Jun (pSer243) in shAnxa11-Hca-P and scramble-Hca-P in 0, 0.01, 0.1 and 1.0 mg/L 5-FU. **B.** Quantified relative levels of c-Jun, c-Jun (pSer73) and c-Jun (pSer243) in shAnxa11-Hca-P *vs* scramble-Hca-P cells. Triplicate independent experiemnts were performed for each assay. * and ** refer to the differences are in statistical significances with *P*<0.05 and 0.01, respectively.

The influence of ANXA11 knockdown on the expression levels of transcriptional factor c-Jun and its phosphorylated form was investigated using WB assay. Slight increase of c-Jun expression level was detected in shAnxa11-Hca-P cells to ANXA11 knockdown compared with scramble-Hca-P cells (Figure 6A), however, the difference was out of statistical significance (Figure 6B, *P*>0.05). The level of c-Jun (pSer73) was significantly increased in shAnxa11-Hca-P than scramble-Hca-P cells (Figure 6B, *P*<0.01). Interestingly, the level of c-Jun (pSer243) decreased (Figure 6A) in shAnxa11-Hca-P cells following ANXA11 downregulation (Figure 6B, *P*<0.05). These data suggest that Anxa11 regulates the malignant properties of Hca-P cells *via* c-Jun path.

ANXA11 is involved in the chemosensitivity of Hca-P to 5-FU *via* c-Jun path. Compared with scramble-Hca-P cells, the expression levels of c-Jun increased in shAnxa11-Hca-P cells following 5-FU administrations with larger upregulation extent than c-Jun in Hca-P cells in responding to ANXA11 downregulation (Figure 6B). More importantly, the expression level difference of c-Jun between shAnxa11-Hca-P and scramble-Hca-P cells (Figure 6A) at the concentrations of 0.1 and 1 mg/L 5-FU were of statistical significances (*P*<0.01, Figure 6B). The level of c-Jun (pSer243) was also detected decreased significantly (Figure 6A and 6B, *P*<0.05) in shAnxa11-Hca-P than scramble-Hca-P cells, while, the downregulation extent was constant and comparable with its downregulation extent in scramble-Hca-P following ANXA11 knockdown. Downregulation of c-Jun (pSer243) is induced by lower level of ANXA11 and not related to 5-FU treatment. c-Jun (pSer73) was found significantly upregulated in shAnxa11-Hca-P cells. Although comparable upregulation extents were determined for c-Jun (pSer73) levels in both shAnxa11-Hca-P and 0.01 mg/L 5-FU-treated-shAnxa11-Hca-P cells, its upregulation extents were calculated in shAnxa11-Hca-P with the treatments of 5-FU at 0.1 and 1.0 mg/L as ~1.48- and 1.50-folds of those in shAnxa11-Hca-P cells in comparisons with the corresponding controls (Figure 6B). ANXA11 knockdown and 5-FU administration seemed to show synergetic effect on c-Jun (pSer73) expression level (Figure 6B). It can be concluded ANXA11 downregulation interferes the properties of Hca-P through upregulating c-Jun (pSer73) and downregulating c-Jun (pSer243). These data suggest that Anxa11 is linked to lymphatic metastasis and 5-FU chemoresistance of Hca-P cells through c-Jun pathway.

## DISCUSSION

As a member of annexin family, Anxa11 is essential to cell cycle, differentiation, apoptosis, vesicle trafficking and signal transduction [6, 7, 40]. It is linked to the autoimmune diseases and cancers [14, 16, 17, 41–43]. Anxa11 was involved in the LNM of colorectal cancer [14], the drug-resistant capacity of ovarian cancer cells or in the prognosis of ovarian cancer patients [16, 44]. This study aimed to investigate the role of Anxa11 playing in LNM and drug resistance with the underlying action mechanisms for murine hepatocarcinoma cell. ANXA11 shows suppressor effect on the malignant behaviours of Hca-P cells. The stable knockdown of ANXA11 by RNAi (Figure 1) promoted the migration (Figure 3A1 and 3B), invasion (Figure 3A2 and 3B) and LN adhesion capacities (Figure 3C and 3D) of Hca-P cells *in vitro* as well as the tumorigenicity and LNM potentials (Figure 4) of Hca-P cells *in vivo*. In addition, ANXA11 knockdown increased the chemoresistance of Hca-P cells to 5-FU (Figure 5A) instead of cisplatin (Figure 5B). Current work indicates Anxa11 is closely associated with the LNM and 5-FU resistance of Hca-P cells, which suggests its potential application as an indicator for the lymphatic malignancy and chemoresistance of HCC.

Cancer cell survival is an important step of its metastasis [45]. The Bcl-2 families play important regulatory roles in cell apoptosis, acting either as the activator (Bax) or the inhibitor (Bcl-2). The balance between the levels of Bcl-2 and Bax is critical for cancer cell survival. Increased Bax/Bcl-2 ratio contributes to cell apoptosis *via* caspase activation through mitochondrial pathway [46, 47]. Our work indicated that ANXA11 downregulation showed no affect on apoptosis of Hca-P cells without interrupting the Bax/Bcl-2 expression ratio and the expression levels of cleaved PARP (Figure 2). The deregulation of ANXA11 also showed on effects on the protein levels of caspase-3, caspase-8, caspase-9 and pro-caspase-8 (unshown data).

c-Jun pathway is linked to the growth, survival, metastasis, invasion and chemoresistance of a variety of cancer cells [21, 48, 49]. Phosphorylations of c-Jun at Ser73 [c-Jun (pSer73)] and Ser243 [c-Jun (pSer243)] are crucial for c-Jun transcriptional activity. Controversially, c-Jun (pSer73) increases, while, c-Jun (pSer243) inhibits [20] the transcriptional activity of c-Jun. c-Jun and JNK pathways were associated with the proliferation, migration and metastasis of HCC [5, 23, 24]. This work established the relationship between Anxa11 and c-Jun in lymphatic metastasis and 5-FU chemoresistance capacities of murine hepatocarcinoma Hca-P cells.

Although without affecting the cell apoptosis of Hca-P cells, ANXA11 stable knockdown (Figure 1) leads to enhanced *in vitro* migration and invasion capacities (Figure 3), *in situ* LN adhesion potential (Figure 3) and *in vivo* malignancy and LNM (Figure 4) of Hca-P cells. ANXA11 downregulation interferes the properties of Hca-P through upregulating c-Jun (pSer73) and downregulating c-Jun (pSer243). Following the downregulation of ANXA11, the expression level of c-Jun (pSer73) was significantly increased while the level of c-Jun (pSer243) was decreased in Hca-P cells (Figure 6). As the upregulation of c-Jun (pSer73) was predominantly evident than the downregulation of c-Jun (pSer243) (Figure 6), which might overally still activates the c-Jun signaling path to promote the *in vitro* and *in vivo* malignant behaviours of Hca-P cells.

ANXA11 knockdown regulates Hca-P chemoresistance to 5-FU *via* c-Jun pathway by up regulating the expression levels of c-Jun and c-Jun (pSer73). Following ANXA11 downregulation in Hca-P cells, the expression level of c-Jun increased in shAnxa11-Hca-P cells against 5-FU administrations at the concentrations of 0.1 mg/L and 1 mg/L 5-FU with statistical significances (*P*<0.01, Figure 6B). The relative expression levels of c-Jun (pSer243) were consistently comparable in shAnxa11-Hca-P cells following ANXA11 knockdown and 5-FU treatments (Figure 6) suggesting Hca-P chemosensitivity to 5-FU is independent of c-Jun (pSer243). c-Jun (pSer73) upregulation extents calculated in shAnxa11-Hca-P with the treatments of 5-FU at 0.1 and 1.0 mg/L were ~1.48- and 1.50-folds of those in shAnxa11-Hca- P compared with the controls (Figure 6B). 5-FU administration exhibits additive promotion on c-Jun (pSer73) expression level in Hca-P cells except for ANXA11 knockdown (Figure 6).

Taken together, current work shows ANXA11 exhibiting tumor suppressor effect on the *in vitro* and *in vivo* malignant behaviors of Hca-P cells. The stable knockdown of ANXA11 enhances the *in vitro* migration and invasion capacities, *in situ* LN adhesion potential, *in vivo* tumor malignancy and LNM, and chemoresistance to 5-FU of Hca-P cells. Anxa11 regulates the malignant properties and chemoresistance of Hca-P cells *via* c-Jun pathway. It is a potential indicator for the malignant progression and a specific therapeutical target of HCC.

## MATERIALS AND METHODS

### Cell culture, animal experiment and ethics statement

Murine hepatocarcinoma Hca-P cell line was maintained by our laboratory. Hca-P cells were grown in Chinese 615-mice (aged 6 weeks, weighting 20±2 g; Certificate of quality number: SCXK (Liao) 2008-0002) abdominal cavity for 7 days. Then the cells were incubated in 85% RPMI 1640 (Gibco, USA) supplemented with 15% fetal bovine serum (FBS: PAA, Australia) in a humidified environment at 37°C with 5% CO_2_.

Mice were provided by the SPF Animal Laboratory Center of Dalian Medical University and treated and sacrificed following the protocols approved by the Experimental Animal Ethical Committee of Dalian Medical University (Permit Number: L2012012).

### Establishment of monoclonal ANXA11 knockdown Hca-P cell line

Targeting shRNAs were designed according to *Anxa11* mRNA sequence (Genbank: NM_ 013469.2) using siDirect and Whitehead. Anxa11-specific shRNA double-stranded oligodeoxyribonucleotides were designed as below: sense: 5′-GCGTGCCAAATCTGTATCCTTT-3′ and antisense:5′-AAAGGATACAGATTTGGCACGC-3′. One shRNA with non-targeting sequence was designed as negative control as below: sense: 5′-GTTCTCCGAACGTGTCACGT-3′ and antisense: 5′-ACGTGACACGTTCGGAGAAC-3′. The expression vectors of shRNA-Anxa11-pGPU6/GFP/Neo and shRNA-scramble-pGPU6/GFP/Neo were constructed according to our previous publication [29, 35, 39]. Transfection was performed with Lipofectamine™ 2000 (Invitrogen, USA) according to manufacturer's protocol. Stably transfected cells were screened against 400 μg/mL G418 for about 14 days, then the monoclonal shRNA-Anxa11-Hca-P (abbreviated as shAnxa11-Hca-P) and shRNA-scramble- Hca-P (abbreviated as scramble-Hca-P) cells were obtained by limited dilution screening.

### Quantitative real-time PCR analysis

Quantitative real-time PCR (qRT-PCR) was used to detect *Anxa11* mRNA level in monoclonal shAnxa11-Hca-P and scramble-Hca-P cell lines. In brief, total RNA was extracted from the cells using Trizol™ reagent (Life technologies, USA). Reverse transcription was performed using PrimeScript™ RT reagent Kit with gDNA Eraser (TaKaRa, Japan). PCR was performed using an Agilent M×3005P real-time PCR machine (Agilent, USA) with FastStart universal SYBR Green Master (ROX) (Roche, Switzerland). PCR primers were listed below: GAPDH, F: 5′-GGTGAAGGTCGGTGTGAACG-3′, R:5′-CTCGCTC CTGGAAGATGGTG-3′; *Anxa11*, F: 5′-GGTC CAACA AGCAGCGTC-3′, R:5′-TCTCAAAGTTTCCCGACAG TTC-3′. The relative mRNA expression levels of *Anxa11* among different group cells were analyzed using 2^−ΔΔCT^ method [50].

### SDS-PAGE and western blotting (WB)

Proteins were extracted using RIPA buffer [10 mM Tris-HCl (pH8.0), 150 mM NaCl, 1% Triton X-100, 0.5% sodium deoxycholate, 0.1% SDS] in the presences of 1 mM Na_3_VO_4,_ 1 μg/mL leupeptin and 0.5 mM PMSF. The supernatants were collected by centrifugation at 12000 rpm for 15 min at 4°C. Protein concentrations were determined by Bradford assay. Then, equal amounts of protein samples were boiled for 5 min in loading buffer, separated by 10% SDS-PAGE and transferred onto NC membrane (Millipore, MA). Being blocked in 5% skim milk (in TBST) for 3 h at RT, the NC membrane was then incubated with primary antibodies shaking with 100 rpm at 4°C overnight. The primary antibodies were GAPDH (1:10000, KangChen, China), ANXA11 (1:1000, Proteintech, USA), Bax (1:1000, Cell Signaling, USA), Bcl-2 (1:500, Cell Signaling, USA), PARP (1:1000; Cell Signaling, USA), Pro-caspase 8 (1:1000, Cell Signaling, USA), c-Jun (1:1000, Sangon, China), phospho-c-Jun (1:1000, pSer73, Sangon, China), phospho-c-Jun (1:1000, pSer243, Sangon, China). The NC membranes were then washed with TBST 3 × 10 min, incubated with secondary antibody conjugated to horseradish peroxidase for 3 h at RT and washed again with TBST buffer 3 × 10 min. Protein bands were visualized by ECL (Advansta, USA) and detected using Bio-Rad ChemiDoc™ MP system (Bio-Rad, USA).

### Cell apoptosis analysis by flow cytometry

The effect of ANXA11 knockdown on Hca-P apoptosis was investigated using Annexin V-FITC/propidium iodide apoptosis detection kit (KeyGEN, China) combined to flow cytometry assay. In brief, 1×10^6^ cells were seeded into a 6-cm dish, incubated at 37°C with 5% CO_2_ overnight and continuously cultured for 24 h. The corresponding Hca-P cells from each group were harvested, washed with ice-cold PBS for 3 times, centrifuged at 1000 rpm for 5 min, resuspended in 500 μL binding buffer, incubated in 5 μL Annexin V-FITC and 5 μL PI in the dark for 30 min at RT. Finally, the cells were immediately subjected to FACSCalibur (BD Biosciences, USA). The results were analyzed using the CellQuest software (BD Biosciences, USA).

### *In vitro* cell migration and invasion assays

Boyden transwell chamber assay was performed to detect ANXA11 downregulation on the migration and invasion capacities of Hca-P cells. For migration assay, 600 μL of RPMI 1640 containing 15% FBS were added into the lower chamber. Then, 5×10^4^ cells from each group in 250 μL serum-free RPMI 1640 were seeded into the upper chambers of transwell inserts with 8 μm pore-size filters and incubated in a humidified incubator at 37°C with 5% CO_2_ for 24 h. The non-migrated cells at the upper surface of filters were swabbed off using cotton swabs. The migrated cells at the lower chamber were fixed by 4% paraformaldehyde for 20 min, stained by 0.1% crystal violet for 20 min, washed with PBS, counted and averaged by selecting 5 random fields per well under a light microscope at a magnification of 100×. Three independent experiments were performed for each assay.

For invasion assay, the 8 μm I.D. polyester membrane fitters’ surfaces of 24-well transwell units were coated with 50 μL ice-cold ECM gel (1:8 dilution with RPMI 1640, Sigma-Aldrich, USA), incubated at 37°C for 3 h and then dried for 30 min at RT. 5×10^4^ cells in 250 μL serum-free RPMI 1640 were then loaded into the upper chamber. The rest procedures were the same as described in migration assay.

### *In situ* cell adhesion potential to lymph node (LN) assay

*In situ* cell adhesion assay was performed to detect ANXA11 downregulation on Hca-P adhesion to LN. Briefly, fresh LNs taken from 615 mice were immediately frozen at −20°C, buried by OCT and sectioned into 10 μm slices. The surfaces of frozen slices of inguinal and axillary LNs were covered in 200 μL serum-free RPMI 1640 containing 1×10^5^ cells from each group. The slices were then incubated at 37°C with 5% CO_2_ for 36 h, washed with ice-cold PBS for 3 times, fixed in 95% ethanol for 5 min, washed with flowing distilled water for 3s and stained with hematoxylin eosin (HE). The number of adherent cells was counted using a microscope (Olympus, Japan) by randomly selecting five fields.

### 5-FU and cisplatin sensitivity assay

Cell counting Kit-8 (CCK-8) assay was performed to detect the influence of ANXA11 knockdown on the chemoresistances of Hca-P cells to 5-FU and cisplatin. Cells from each group were seeded into a 96-well plate at a density of 7×10^3^ cells/well in 100 μL medium and cultured at 37°C with 5% CO_2_ overnight. Then cells were treated with 5-FU with the final concentrations of 0, 0.001, 0.01, 0.1, 1, 10 mg/L, and with cisplatin with the final concentrations of 0, 1, 2, 4, 8 and 16 μM, respectively. Being incubated at 37°C with 5% CO_2_ in 5-FUfor 48 h and cisplatin for 48 h, the viability of the cells from each group was then measured using CCK-8 assay. The absorbances at 450 nm were measured using a microplate reader (Thermo, USA). Cell viability ratio was defined as the absorbance of sample divided by the absorbance of control. Triplicate experiments were performed for each assay.

### Hoechst 33258 staining assay

This assay was performed to investigate stable ANXA11 knockdown on Hca-P apoptosis to 5-FU induction. Cells from each group were treated with different concentrations of 5-FU for 48 h, collected by centrifugation at 1000 rpm, washed with PBS buffer 3 times and fixed in 4% paraformaldehyde for 10 min at 4°C. Cells were then collected by centrifugation at 1000 rpm for 5 min, resuspended and washed with 2 × 200 μL PBS buffer. Then, the cells from each group were stained with Hoechst 33258 working solution in darkness for 10 min at RT, washed with ddH_2_O for 3 times and immediately imaged using an inverted fluorescence microscope (Olympus, Japan) operated with the excitation wavelength of 340 nm at 200×.

### *In vivo* tumorigenicity and LNM assay

The effect of ANXA11 downregulation on the LNM of Hca-P cells was measured using Hca-P-transplanted mouse model. 1×10^6^ cells in 30 μL serum-free RPMI 1640 from each group were subcutaneously inoculated into the left footpads of inbred Chinese 615 mice. Each group had 10 mice with half male and half female. Following 1, 4, 8, 11, 15, 18 and 21 days after inoculation, the volumes of solid tumors formed in mice footpads were calculated according to the equation, V = π/6×length×width×height, where the unit for length, width and height is in mm. In 21 days after inoculation, the mice were sacrificed by collecting their LNs. For LNM analysis, the inguinal and axillary LNs were isolated and fixed in 10% neutral formalin, embedded in paraffin, sectioned into 4 μm slices, stained by HE and examined by a light microscope (Olympus, Japan). LNM rates of each group cells were then obtained.

### Data processing and statistical analysis

SPSS 17.0 software (SPSS Inc., Chicago, IL, USA) was used for all statistical analysis. All experimental date were represented as mean±SD of at least three independent experiments. The significant differences between the groups were analyzed using student's *t* test. Statistical significant differences were labeled as * and ** for *P* values below 0.05 and 0.01.

## Abbreviations

ALG-2: apoptosis-linked gene-2 protein
Anxa11: Annexin A11
AP-1: activator-protein 1
Bax: Bcl-2 associated X protein
Bcl-2: B-cell lymphoma 2
CCK-8: cell counting kit-8
ECL: enhanced chemiluminescent
ECM: extracellular matrix
FBS: fetal bovine serum
FITC: fluorescein isothiocyanate
5-FU: 5-fluorouracil
GAPDH: glyceraldehyde-3-phosphate dehydrogenase
Hca-F: hepatocarcinoma ascites cell F
Hca-P: hepatocarcinoma ascites cell P
HCC: hepatocellular carcinoma
HE: hematoxylin eosin
JNK: Jun-N-terminal kinase
LN: lymph node
LNM: LN metastasis
NC: nitrocellulose
OCT: optimum cutting temperature compound
PARP: poly adeno ribose polymerase
PBS: phosphate buffered saline
PI: propidium iodide
qRT-PCR: quantitative real-time polymerase chain reaction
RIPA: radio-immunoprecipitation assay
RNAi: RNA interference
RPMI: Roswell Park Memorial Institute
RT: room temperature
SD: standard deviation
SDS-PAGE: sodium dodecyl sulfate polyacrylamide gel electrophoresis
shRNA: short hairpin RNA
SPF: specific pathogen free
TBST: tris-buffered saline with Tween-20
WB: Western blotting.
